# Surrogate outcomes: experiences at the Common Drug Review

**DOI:** 10.1186/1478-7547-11-31

**Published:** 2013-12-17

**Authors:** Angela Rocchi, Shoghag Khoudigian, Rob Hopkins, Ron Goeree

**Affiliations:** 1 Axia Research, 2068 Waterbridge Drive, Burlington, ON L7M 3W2, Canada; 2 McMaster University, Hamilton, ON, Canada; 3 PATH Research, Hamilton, ON, Canada

**Keywords:** Reimbursement, Decision-making, Surrogate outcomes, Health technology assessment

## Abstract

**Background:**

Surrogate outcomes are a significant challenge in drug evaluation for health technology assessment (HTA) agencies. The research objectives were to: identify factors associated with surrogate use and acceptability in Canada’s Common Drug Review (CDR) recommendations, and compare the CDR with other HTA or regulatory agencies regarding surrogate concerns.

**Methods:**

Final recommendations were identified from CDR inception (September 2003) to December 31, 2010. Recommendations were classified by type of outcome (surrogate, final, other) and acceptability of surrogates (determined by the presence/absence of statements of concern regarding surrogates). Descriptive and statistical analyses examined factors related to surrogate use and acceptability. For thirteen surrogate-based submissions, recommendations from international HTA and regulatory agencies were reviewed for statements about surrogate acceptability.

**Results:**

Of 156 final recommendations, 68 (44%) involved surrogates. The overall ‘do not list’ (DNL) rate was 48%; the DNL rate for surrogates was 41% (p = 0.175). The DNL rate was 64% for non-accepted surrogates (n = 28) versus 25% for accepted surrogates (odds ratio 5.4, p = 0.002). Clinical uncertainty, use of economic evidence over price alone, and a premium price were significantly associated with non-accepted surrogates. Surrogates were used most commonly for HIV, diabetes, rare diseases, cardiovascular disease and cancer. For the subset of drugs studied, other HTA agencies did not express concerns for most recommendations, while regulatory agencies frequently stated surrogate acceptance.

**Conclusions:**

The majority of surrogates were accepted at the CDR. Non-accepted surrogates were significantly associated with clinical uncertainty and a DNL recommendation. There was inconsistency of surrogate acceptability across several international agencies. Stakeholders should consider collaboratively establishing guidelines on the use, validation, and acceptability of surrogates.

## Background

In 2003, the Common Drug Review (CDR) was created to provide a single national process to review the comparative clinical evidence and cost-effectiveness of new drugs, and to make formulary listing recommendations to Canadian publicly funded federal, provincial and territorial drug benefit plans (excluding Quebec) [1]. A centralized review process was intended to mitigate inconsistencies which existed across jurisdictions, both in drug review expertise and drug access. The CDR process and the requirements for submission are outlined in detailed documents available at http://www.cadth.ca[2,3].

A recent Canadian publication examined trends and predictors in CDR recommendations. Under logistic regression, four factors were found to be significantly associated with a ‘do not list’ (DNL) recommendation: a statement of clinical uncertainty, a request for reconsideration (a form of appeal), the use of price as the only economic evidence, and price greater than comparators [4]. A comparison of recommendations from Canada, Australia and the UK also found that clinical uncertainty was a key issue, with uncertainty typically arising from inadequate study design, inappropriate comparators or unvalidated surrogate endpoints [5]. That study concluded that all three agencies “face common issues with respect to the quality and strength of experimental evidence in support of a clinically meaningful effect”. Further, chairs of the CDR’s expert advisory panel have noted that clinical uncertainty, and specifically surrogate outcomes, remain one of their greatest challenges [6].

Surrogate outcomes are defined as a laboratory measurement or a physical sign used as a substitute for a clinically meaningful endpoint that measures directly how a patient feels, functions or survives, and that is expected to predict the effect of the therapy [7]. Surrogates are often biomarkers such as hemoglobin A1C [HbA1c], blood pressure, lipid levels, etc. Surrogate outcomes are used in clinical trials for reasons of efficiency and practicality; they can be measured with fewer patients, less invasiveness and a shorter observation period [8]. Where surrogate outcomes have validated links with final endpoints, their use can greatly facilitate clinical research. However, in the absence of validated links, there can be uncertainty about patient benefit; and even where the epidemiologic basis is sound, long-term safety and other unanticipated issues may predominate [9]. For example, while blood pressure is conclusively linked to cardiovascular morbidity and mortality, antihypertensive drugs do not necessarily reduce morbidity or mortality as expected [10].

In contrast, a final outcome produces an end unit of health effect: survival, cure, or prevention of such an event (such as pregnancy or infection). For therapeutic areas where mortality or cure is not a relevant measure, clinical endpoints and scales measure how a patient feels or functions, but not survives (such as disability scores, depression scales, psoriasis severity, arthritic joint counts, incontinence episodes, etc.).

Surrogate outcomes have become fundamental to drug development, but their use in regulatory decision-making is regularly questioned when large observational studies reveal unpredicted mortality from effective drugs. Regulatory agencies such as the United States’ Food and Drug Administration (FDA) have supported the formal inclusion of surrogate outcomes in the regulatory process. The FDA’s Critical Path Initiative identified surrogate outcomes as an important opportunity to evaluate and predict the safety, effectiveness, and manufacturability of medical products [11]. At the same time, the FDA has also instituted a requirement for evaluating the cardiovascular risk of new diabetes drugs, based on unexpected cardiovascular events among diabetic patients treated with rosiglitazone, whose metabolic effects were expected to reduce, rather than increase, such events [12,13].

Beyond regulatory approval, health technology assessment (HTA) agencies also review evidence based on surrogate outcomes. In contrast to the US regulator, HTA agencies are often cautious about the use of surrogate outcomes, and in many cases have developed methodological guidelines for the use and validation of surrogate outcomes [14]. Furthermore, for HTA agencies that review cost-effectiveness data, surrogates are often used as the basis for cost-effectiveness research [15]. HTA agencies are dependent on the drug development evidence base as influenced by the regulator. These two different audiences may have different informational needs [16]. Regulators may be more focused on safety and shorter-term efficacy and registration trials are specifically designed to answer these questions, usually with guidance from regulatory agencies. HTA agencies may focus on a longer-term perspective, and the occurrence or prevention of downstream ‘hard’ events. These may not be addressed within the registration trial data package or the focus on short-term outcomes. This may result in different perspectives on the acceptability of surrogates, which could be revealed by a comparison of the two types of agencies.

While the CDR does not have a formal position or guideline on the use of surrogate outcomes, some recommendations have included statements that show concern for the use of surrogates and their relationship to patient benefit. These statements of concern have varied both within and between drugs and therapeutic areas. It is important to investigate whether surrogate concerns may lead to higher rates of clinical uncertainty, and thus impact the evaluation drugs in therapeutic areas where surrogate use is common.

The objectives of this research were to identify factors associated with surrogate use and acceptability at the CDR, and to compare the CDR with other HTA or regulatory agencies regarding surrogate concerns.

## Methods

### Databases

The CDR analysis constituted all published recommendations from inception (September 2003) to December 31, 2010. ‘Final Recommendations and Reasons for Recommendation’ were retrieved from the CADTH website (http://www.CADTH.ca). The same drug could have had multiple reviews and recommendations. For drugs that had multiple submissions for the same indication, qualitative statements about surrogates were abstracted from all recommendations, although only the most recent recommendation was used to determine the final listing recommendation. For example, insulin detemir was initially reviewed by CEDAC in June 2005 for the treatment of Type 1 and 2 diabetes mellitus. A resubmission for the same indication (based on new information) was reviewed in July 2006. The final listing recommendation was determined from the 2006 resubmission, but qualitative statements about surrogates could be drawn from either submission. In contrast, subsequent submissions for previously-reviewed drugs that were based on new indications were considered as separate, individual recommendations. For example, insulin detemir was resubmitted in 2009 for a new indication (Type 1 diabetes in pediatric patients). This second indication was included in the dataset as a separate, unique submission.

The CDR has multiple types of recommendations. ‘Do not list’ was a negative recommendation; all others were considered a positive recommendation (‘list’, ‘list in a similar manner to other drugs in the same class’ and ‘list with conditions/criteria’).

For the international comparison of the CDR to other agencies, rather than investigating all 68 drug submissions which involved a surrogate, a convenience sample was selected of submissions from therapeutic areas where surrogate use was common, surrogate acceptability was questioned, and the same surrogate was used across multiple submissions (at least three drugs in each therapeutic area in order to have a reasonable sample). All submissions from three therapeutic areas were examined: type 2 diabetes oral drugs (3 drugs), hepatitis (5 drugs) and pulmonary arterial hypertension (5 drugs). Therapeutic areas where surrogate use was unquestioned were excluded from the international comparison. For example, HIV drugs and insulin therapies were excluded because the CDR never stated any concerns about the surrogate outcomes as used in these areas (viral load and HbA1c respectively). Others (rare diseases, cancer) were impractical to investigate as a group because there was too much disease heterogeneity within the category and limited use of the same surrogate across submissions.

The HTA agencies selected for comparison included the English-language central review agencies which post detailed recommendations and to which the CDR is regularly compared: Australia’s Pharmaceutical Benefits Advisory Committee (PBAC), Scotland’s Scottish Medicines Consortium (SMC) and England’s National Institute for Health and Clinical Excellence (NICE) [5,16,17]. The regulatory agencies were: Health Canada (to compare with the CDR), European Medicines Agency (EMA, to compare with the SMC and NICE) and the Food and Drug Administration (FDA, as the largest and most influential single regulatory agency). For these agencies, websites were searched to determine the initial and all subsequent reports and recommendations on the indications under consideration by the CDR: the ‘Summary Basis for Decision’ from Health Canada, the reviewers reports (‘Medical’ ‘Clinical’ or ‘Summary’) from the Centre for Drug Evaluation and Research for the FDA, and the ‘Assessment Report’ or ‘CHMP Assessment Report’ for the EMA.

### Data abstraction

A database of CDR submissions was developed initially using the information available in the public recommendations. Data were abstracted for variables under four distinct categories of inquiry: submission specifics, drug characteristics, clinical evidence, and economic evidence. All variable responses had formal definitions, developed by the abstractors. Any database disagreements were resolved by discussion between the two abstractors. Full details of the methodology to establish the CDR database are found in an earlier publication [4].

For a number of variables, particularly for the clinical and economic evidence, there was insufficient and/or inconsistent information available in the public documents. As a result, some response definitions reflected whether information was stated or identified, with the alternate response option being ‘not stated’ or ‘not identified’. For example, clinical uncertainty required an explicit statement that the clinical evidence was ‘unknown’ ‘uncertain’ ‘unproven’ ‘insufficient’ or ‘not sufficient’. If a definitive statement regarding clinical uncertainty was not present, then the response assigned was ‘not identified’. Similarly, outcomes were defined as ‘not acceptable’ only if there was an explicit statement of such. There were no explicit statements that the chosen outcome was acceptable. Therefore, for all other outcomes, their acceptability was ‘not identified’.

For the current analysis, the original CDR database was extended by adding the type of outcome: surrogate, final or ‘other’ (clinical endpoints and scales). A surrogate outcome was defined as a biomarker intended to substitute for a clinical endpoint, such as: HbA1C, viral load, 6 minute walk distance (6MWD), blood pressure, lipid levels, forced expiratory volume, intraocular pressure, and biochemistry. Surrogate outcomes were further classified into ‘accepted’ or ‘not accepted’ using the methodology above – that there had to be an explicit statement of concern about the surrogate, or a stated preference for alternative outcomes. All other responses and non-responses were classified by default as ‘accepted’. Note, for an ‘accepted’ surrogate, there could be explicit negative statements about other aspects of the clinical use of the outcome (such as study design issues), but not about the outcome itself. This binary system of classification was used for the analysis that was limited to CDR recommendations.

Final outcomes were limited to: death, cure or prevention of event (e.g. pregnancy). All other outcomes were defined as ‘other’ and constituted clinical endpoints or scales. For some therapeutic areas (such as mental health, neurology, or analgesia), tools such as endpoints or scales were the appropriate measures of disease status. These tools ideally would be objective, reproducible and with minimal measurement bias. Examples include: ACR 20 for assessment of rheumatology diseases, EDSS for assessment of multiple sclerosis, or HAM-D for major depressive disorder.

All drugs in this analysis were approved in Canada under routine drug development pathways. Only a small minority of drugs (7%) were approved based on phase II trials. It was reasonable to assume that all clinical trial outcomes were reviewed by appropriate regulatory agencies, and that clinical experts agreed the trial outcomes represented appropriate, valid measurements of disease status and drug efficacy for phase IIIA drug development trials.

For the international comparison, two researchers (AR, PD) searched each agency website to obtain relevant documents and identify sections within each document that concerned surrogates, while both researchers abstracted data and resolved all disagreements and discrepancies. For each drug, statements regarding surrogates were examined and classified under ten levels of acceptability (Table 1). CDR recommendations were re-reviewed and re-classified using this more extensive system for the international comparison (replacing the binary classification used in the CDR-only analysis). Under either system, however, it remained true that there had to be explicit statements to support lack of acceptability. Supporting statements to justify the classification were recorded in the database. Other abstracted data included: date of review, funding recommendation, reimbursement criteria, and preferred outcomes (if stated). If available, national guidelines were obtained and compared to the funding recommendation as well as the qualitative surrogate comments.

**Table 1:** Classification system for surrogate acceptability

Explicit yes = yes (e)		Statement that the surrogate was accepted, valid, established or clinically relevant
Implicit yes*	‘Used before’ = yes (ref)	Statement that the surrogate has been used for other, earlier drugs in this indication
	‘Guidelines’ = yes (guid)	Statement that the surrogate is identified in guidelines as appropriate
	‘Evidence 1’ = yes (e1)	Statement that there is some evidence linking the surrogate to the final outcome
	‘Evidence 2’ = yes (e2)	Statement that there is some evidence linking the drug to the final outcome
No statement = (N/S)		No qualitative statement whether the surrogate was acceptable or not
Not applicable = (N/A)		No review was conducted or available
Implicit no	‘Reference’ = no (ref)	Statement of reference to other (preferred) outcomes but no direct comment on the surrogate
	‘Evidence 2’ = no (e2)	Statement that there is no evidence linking the drug to final outcomes
Explicit no	‘Evidence 1’ = no (e1)	Statement that there is no evidence linking the surrogate outcome to final outcomes

*Could additionally record a statement of concern.

Some reviews included both supportive and critical statements about a surrogate. For a review that resulted in a negative recommendation, it was assumed that the critical statements were more representative of the agency’s views on the surrogate. In this case, the surrogate was assigned to one of the non-accepted categories. For a review that resulted in a positive recommendation, it was assumed that the supportive statements were more representative of the agency’s views on the surrogate. In this case, the surrogate was assigned one of the ‘accepted’ levels, but a statement of concern was noted.

### Statistical analysis

Every unique recommendation was considered a separate observation. Once all recommendations were categorized, descriptive statistics were performed, to identify characteristics associated with surrogates overall and with accepted versus non-accepted surrogates. The focus was on the recommendation status (DNL versus list) and the factors that were previously proven to be associated with a DNL.

Differences across HTA agencies were descriptively presented as Yes/No indications of statements concerning surrogate acceptability for the drugs included in the analysis.

#### Factors associated with surrogate use

Drug recommendations and all factors significantly associated with a DNL in the previous publication by Rocchi et al. [4] were individually tested for independence. These variables were tested against the three type of outcomes (final, others, surrogates) using chi-square tests with degrees of freedom of (rows-1)×(columns-1) and a level of significance (α) of 0.05. When a variable was rejected for independence (p < 0.05), a statistically significant association was determined to be present between the tested variables.

#### Factors associated with surrogate acceptability

In the binary univariate logistic analysis, a regression with surrogate acceptability as the dependent variable (not accepted = 1, and accepted = 0) was run against variables that were associated with DNL in the study conducted by Rocchi et al. [4] and against other covariates such as first in class, first in disease, life threatening disease and priority review requested. An OR > 1 indicated that the presence of a factor (e.g. clinical uncertainty, higher price, etc.) was statistically associated with non-accepted rather than accepted surrogates [18]. Associations were significant at p < 0.05.

## Results

### Dataset disposition

From inception to December 31, 2010, there were 156 final unique recommendations. Of these, there were 68 surrogate outcomes (44%), 26 final outcomes (17%), and the remaining 62 (44%) were ‘other’ (clinical endpoints and scales). Of the 68 surrogate outcomes, 28 were explicitly defined as ‘not accepted’ (41% of the surrogate outcomes). The remaining 40 drugs (59%) did not have any explicit negative statements and were classified by default as ‘accepted’.

### CDR analysis

#### Reimbursement recommendation

Of the 156 recommendations under review, there were 77 DNL recommendations, for an overall 48% DNL rate. Table 2 depicts the results of the CDR analysis.

**Table 2:** Percentage of drug recommendations based on outcome

**Factor**	**Final outcome**^**¥ **^**(n = 26)**	Other outcome* (n = 62)	**Surrogate outcome**^**§ **^**(n = 68)**
DNL recommendation	15/26 (58%)	32/62 (52%)	28/68 (41%)
Statement of clinical uncertainty	13/26 (50%)	36/62 (58%)	26/68 (38%)
Price only economic factor	12/26 (46%)	30/62 (48%)	34/68 (50%)
Economic considered	11/26 (42%)	29/62 (47%)	28/68 (41%)
Price greater than comparators	19/26 (73%)	30/62 (48%)	29/68 (43%)

^¥^Final outcome = end unit of health effect.

*Other = clinical endpoints and clinical scales.

^§^Surrogate outcome = biomarker intended to substitute for a clinical endpoint.

There was a gradient of approval based on type of outcome: final outcomes were associated with the highest DNL rate at 58%, followed by ‘other’ at 52%. Surrogate outcomes had the lowest rate of rejection at 41%. These differences were not statistically significant.

The DNL rate was significantly different based on surrogate acceptability. For non-accepted surrogates, the DNL rate was higher at 64%; for accepted surrogates, the DNL rate was lower at 25% (odds ratio [OR] of a DNL for a non-accepted versus an accepted surrogate: 5.4, p = 0.002, Tables 2 and 3).

**Table 3:** Analysis of the drug recommendations with surrogate outcomes

**Factor**	**Descriptive analysis**		**Univariate logistic regression analysis based on “not-accepted” surrogates**
	**Accepted surrogates**^**¥ **^**N = 40**	**Non-accepted surrogates**^**§ **^**N = 28**	**Odds ratios**^**∞ **^(95%CI) [p value]*
***Factors Associated with a DNL***			
DNL recommendation	10/40 (25%)	18/28 (64%)	5.4 (1.9-15.5) **[p = 0.002]**
Clinical uncertainty	8/40 (20%)	18/28 (64%)	7.2 (2.4-21.5) **[p < 0.001]**
Price Only	28/40 (70%)	6/28 (21%)	0.1 (0.0-0.4) **[p < 0.001]**
Economic considered	11/40 (28%)	17/28 (61%)	4.1 (1.4-11.4) **[p = 0.007]**
Price greater than comparators	13/40 (33%)	16/28 (57%)	2.8 (1.0-7.5) **[p = 0.046]**
***Other Factors***			
First in class	6/40 (15%)	14/28 (50%)	5.7 (1.8-17.7) **[p = 0.003]**
First in disease	2/40 (5%)	5/28 (18%)	4.1 (0.7-23.1) [p = 0.106]
Life threatening	3/40 (8%)	9/28 (32%)	5.8 (1.4-24.1) **[p = 0.015]**
Priority review requested	7/40 (18%)	9/28 (32%)	2.2 (0.7-7.0) [p = 0.166]

^**∞**^; Odds Ratio > 1 is associated with higher odds of the surrogate not being accepted given the presence of a factor.

*Bold p values indicate statistical significance under univariate analysis.

^¥^Accepted surrogates = all the recommendations with surrogate acceptability classified as: yes (e1) = implicit yes “evidence 1”; yes (e2) = implicit yes “evidence 2”; yes (used) = implicit yes “used before”; yes (ref) = implicit yes “reference”; yes (e) = explicit yes; N/S = no statement; N/A = not applicable.

^**§**^Not-accepted surrogates = all the recommendations with surrogate acceptability classified as: no (e2) = implicit no “evidence 2”; no (ref) = implicit no “reference”; no (e) = explicit no “evidence 1”; no (e1 + e2) = explicit no “evidence 1” and implicit no “evidence 2”.

#### Factors associated with surrogate acceptability

The distribution of clinical uncertainty mirrored the reimbursement recommendation closely, as was demonstrated in the original CDR analysis [4]. Clinical uncertainty was most likely to be present for submissions using ‘other’ outcomes (58%; p = 0.999), followed by submissions using final outcomes (50%; p = 0.062). Clinical uncertainty was least likely to be present for submissions using surrogate outcomes (38%; p = 0.045).

If the surrogate was assigned ‘acceptable’, then clinical uncertainty was present for only 20% of submissions. In contrast, 64% of submissions with non-accepted surrogates had clinical uncertainty (OR of clinical uncertainty for a non-accepted versus an accepted surrogate: 7.2, p < 0.001, Tables 2 and 3).

Consideration of economic evidence was categorized as ‘clinical undetermined’ (no consideration of economic evidence including price), ‘economic considered’ (where there was mention of an economic model or cost-effectiveness) and ‘price only’. The distribution between ‘economic considered’, ‘price only’ and ‘clinical undetermined’ was quite consistent across the three types of outcomes. However, within the category of surrogates, differences emerged. For the majority (70%) of accepted surrogates, the discussion involved only price. This suggested a simplified decision-making process for these cases: drugs were deemed equivalently efficacious and similarly priced. The odds ratio for using price only for non-accepted versus accepted surrogates was 0.1 (p < 0.001, Table 3), confirming that recommendations with price as the only economic evidence are less likely to have concerns regarding the surrogate. For the majority (61%) of non-accepted surrogates, the discussion considered economic evidence in the form of a model or cost-effectiveness results – suggesting a more complex consideration of a product’s value (OR for economic evidence with a non-accepted versus accepted surrogates: 4.1; p = 0.007, Table 3).

Price was categorized into a binary variable: (1) greater than all CEDAC-identified comparators or at the same price as the most expensive comparator cited by CEDAC, and (2) all other responses. For accepted surrogates, price was greater than comparators for only 33% of submissions, but price was greater almost twice as often for non-accepted surrogates (57%, OR for price greater than comparator for non-accepted versus accepted surrogates: 2.8; p = 0.046, Table 3).

The fourth factor which was significantly associated with a DNL was a request for reconsideration, a manufacturer choice that is unrelated to the clinical or economic characteristics of a drug and consequently was not reported in this analysis.

#### Therapeutic area

**Figure 1:**
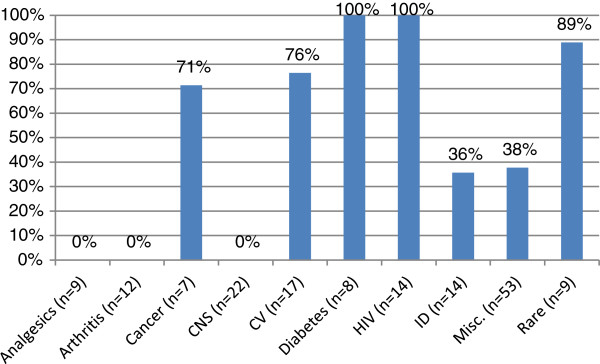
**Percentage of drug recommendations with surrogate outcomes by therapeutic area.** Y axis: Percent of recommendations using surrogate outcomes. X axis: Therapeutic area.

**Figure 2:**
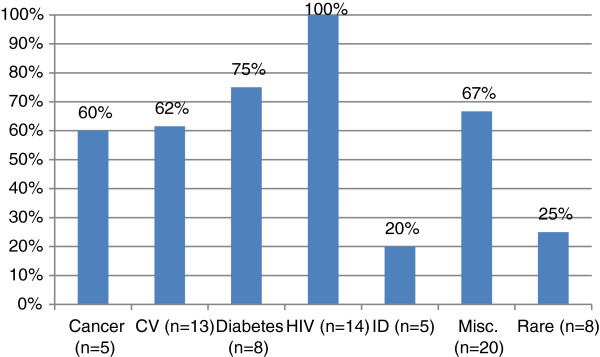
**Percentage of drug recommendations with surrogate acceptability by therapeutic area.** Y axis: Percent of surrogate outcomes with acceptability. X axis: therapeutic area. CV = cardiovascular; HIV = human immunodeficiency virus; ID = infectious disease; Misc. = miscellaneous. Non-accepted surrogates for CV: 4/5 in pulmonary arterial hypertension; ID: 4/4 in hepatitis B; diabetes: 2/2 were oral antidiabetic agents. Rocchi et al. Surrogate Outcomes at the CDR.

The use of different types of outcomes was not randomly distributed across therapeutic area (Figure 1), nor was surrogate acceptability (Figure 2). For example, ‘other’ outcomes were used exclusively in submissions for analgesics and arthritis drugs (such as pain scores, ACR20, etc.) – therapeutic areas that rely exclusively on clinical endpoints and scales. On the other hand, surrogate outcomes were used exclusively for HIV antiretrovirals and diabetes drugs. In HIV, viral load was accepted in every submission, and these drugs were also associated with a 100% acceptance rate (0% DNL rate). In diabetes, HbA1C was used universally. For type 1 diabetes, HbA1C was never questioned as an appropriate outcome for insulin therapy, but it was non-accepted for two of three type 2 oral diabetes drugs. Surrogate outcomes were also used frequently (89%) with drugs for rare diseases, since the natural history of these diseases is usually not well elucidated. Surrogates such as blood pressure and lipid levels were common in cardiovascular disease (76% of submissions). Infectious disease was split between acute infections which could employ final outcomes (cure rates) for 64% of these submissions, and chronic infections where the time horizon of downstream outcomes required use of surrogates (36% of submissions: hepatitis B and C).

#### Other factors

Compared to accepted surrogates, non-accepted surrogates were significantly more likely to be first-in-class (50% versus 15%, OR 5.7; p = 0.003) and for life-threatening diseases (32% versus 8%, OR 5.8; p = 0.015). Non-accepted surrogates were more frequently first for disease drugs (18% versus 5%, OR 4.1; p = 0.106), and requested for priority review (32% versus 18%, OR = 2.2, p = 0.166) but these differences were not significant.

### Agency comparison

**Figure 3:**
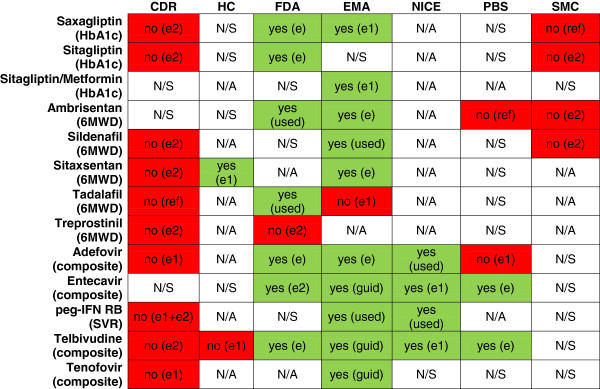
**Comparison of international agencies: concerns with surrogate outcomes.** Y axis: Drug submission. X axis: Agency. *No: no (e2) = implicit no “evidence 2”; no (ref) = implicit no “reference”; no (e) = explicit no “evidence 1”; no (e1 + e2) = explicit no “evidence 1” and implicit no “evidence 2”; Yes: yes (e1) = implicit yes “evidence 1”; yes (e2) = implicit yes “evidence 2”; yes (used) = implicit yes “used before”; yes (ref) = implicit yes “reference”; yes (e) = explicit yes; Not identified: N/S = no statement; N/A = not applicable; Red shades = negative statements of surrogate acceptability; Green shade = positive statement of surrogate acceptability; HbA1c = hemoglobin A1c; 6MWD = 6 minute walk distance; composite = histology, virology, serology; SVR = sustained virological response; CDR = Common Drug Review; HC = Health Canada; FDA = Food and Drug Administration; EMA = European Medicines Agency; NICE = National Institute for Health and Clinical Excellence; PBS = Pharmaceutical Benefit Scheme; SMC = Scottish Medicines Consortium.

Figure 3 depicts the results of the comparison between agencies, with acceptance of surrogates depicted using a red-green colour scheme (red for non-accepted surrogates, green for accepted surrogates). The thirteen drugs that were included in this analysis were chosen based on the criteria that the CDR expressed concerns with the outcomes used for almost all drugs in these therapeutic areas. Predictably, therefore, there was a high level of lack of acceptability at the CDR (77% of recommendations).

Not all drugs were reviewed by all HTA agencies, depending on their respective mandates. In particular, while the CDR receives submissions for almost every new chemical entity for outpatient use in Canada, NICE does not – and in fact only reviewed five of the thirteen drugs. NICE rendered four ‘implicit acceptance’ of the surrogates used for hepatitis drugs, with the fifth as ‘no statement’. PBAC and SMC generally avoided qualitative statements about surrogates (for 64% of PBAC reviews and 60% of SMC reviews). Of the submissions that included qualitative statements about surrogates, these were often negative (two of four for PBAC and four of four for SMC).

Regulatory agencies often provided explicit statements of acceptance of the surrogate outcome (83% of EMA reviews and 64% of FDA reviews). Negative statements were rare at regulatory agencies, with only one for each agency. Health Canada had the least accessible reviewers’ reports with which to inform the analysis.

## Discussion

The CDR embraces an evidence-driven process that relies on comparative effectiveness, cost-effectiveness and price, while simultaneously weighting multiple factors. As with similar central HTA agencies, clinical uncertainty is critical to the discussion. This research has shown that surrogate acceptability was closely aligned with clinical uncertainty – a predictable association, but important to establish as a preface to more in-depth analysis. An unaccepted surrogate, clinical uncertainty and a DNL recommendation was a common triad – which seems entirely reasonable and appropriate. Often, there was also a higher price than comparators, raising the importance of an economic model to determine incremental value.

This research has also shown that submissions based on surrogates were more likely to receive a positive recommendation. A well-accepted surrogate, such as HIV DNA viral load, can facilitate rapid and efficient clinical trials, regulatory approval and comparative evaluation by HTA agencies. Accepted surrogate outcomes were associated with a low DNL rate of 25%. Surrogate acceptance was more common when price was commensurate with comparators: the bar for clinical certainty, and the acceptability of a surrogate, is partially set by the price of the new intervention.

The Rocchi et al. analysis of CDR recommendations found that there were statistically significant differences in DNL rates between therapeutic areas [4]. Similarly, this analysis found a highly non-random distribution of types of outcomes across therapeutic areas. The concern is that any systematic lack of preference for surrogate outcomes could lead to high barriers to access for the therapeutic areas that rely on surrogate outcomes.

It was a challenge to identify reasons for surrogate acceptability. An obvious reason was a relative lack of epidemiologic evidence to link surrogates with final outcomes, or drugs to final outcomes – typically due to a long-term horizon for such events (hepatitis B) or the challenge of studying infrequent events or small populations (drugs for rare diseases). In contrast, excellent epidemiological data confirmed that antiviral drugs which reduce HIV DNA viral loads to negligible levels will dramatically reduce HIV mortality [19]. (Note that viral load is the same surrogate outcome that is considered inadequate for hepatitis B). Outside of epidemiology, however, there was considerable variation in the acceptability of the same surrogate between different drug submissions. Unknown potential long-term safety issues with new classes of drugs could shift the perception of an otherwise-accepted surrogate, eliciting a preference for final outcome studies to confirm that the expected improvements in efficacy also improved overall safety (such as new drug classes of antihypertensives or oral diabetic agents). Evolutions in clinical practice could change the acceptability of a surrogate, either with the evaluation of more recent drugs, or on resubmission of an earlier drug. Premium pricing was noted above as another reason for variation in acceptability for the same surrogate.

Oral diabetes drugs were an interesting example of multiple surrogate-related issues. HbA1c is recognized in international clinical practice guidelines as the relevant treatment goal and primary outcome for clinical trials [20,21]. HbA1c was never raised as a concern for insulins at the CDR, whether they were used for Type 1 or Type 2 diabetes. However, HbA1c was rejected as a surrogate for an oral Type 2 antidiabetic agent (sitagliptin). At the time, conflicting epidemiologic studies were published about the benefits of aggressive HbA1c reduction in Type 2 diabetics with long-standing cardiovascular disease. Beyond the epidemiologic confusion about HbA1c, the submissions involved a new class of drugs, with the attendant long-term safety concerns. The new drugs entered the oral diabetes market with prices higher than the existing, older and genericized products. The decline in utilization of another oral drug class (the glitazones) left a clinical vacuum that eventually increased pressure for access to alternative agents.

For the selected drugs studied in the international comparison, other international HTA agencies less frequently stated concerns about the use of surrogates. In most cases, there was no comment on the surrogate at all from HTA agencies (which is not necessarily an endorsement of the surrogate). The sample was deliberately selected from submissions for which the CDR had voiced concerns, which was a clear bias. However, these were therapeutic areas for which outcomes were clearly a challenge: there was considerable consternation across agencies regarding hepatitis drugs because of the long horizon for downstream outcomes, and with respect to pulmonary arterial hypertension drugs, because of uncertain value of improved exercise tolerance and any putative relationship to overall survival. Comments from other agencies focused more on the complex and challenging nature of clinical research in these therapeutic areas, rather than specific concerns about the use of a specific surrogate. Possible explanations for discrepancies between HTA agencies could include: different mandates, different legislative environments, different expert panel composition, different normative expectations around evidence, and/or different risk tolerances around extrapolation of evidence.

Regulatory agencies had few concerns, and in fact often had positive statements of acceptance about surrogate outcomes. Note however that the body of clinical evidence developed by manufacturers at the time of launch is intended specifically to meet the informational needs of a regulatory agency (and not necessarily a reimbursement or HTA agency). Consequently, the use of surrogate outcomes can be entirely appropriate for these agencies, particularly to support intermediate treatment objectives specified in a product monograph, such as ‘regulation of blood glucose’ or ‘improvement in exercise capacity’.

The same drug file, presumably based on the same pivotal clinical trials, could generate statements ranging from an explicit acceptance to an explicit rejection (e.g. adefovir, telbivudine). Statements regarding HbA1c for sitagliptin ranged from ‘a very well accepted surrogate…[which] predicts both microvascular and macrovascular complications’ by the FDA to the CDR: ‘there is an absence of direct evidence on whether sitagliptin reduces micro or macro vascular outcomes and the relationship between HbA1c and vascular outcomes may differ for new drug classes’. These opposing comments may be due to the examination of a different evidence base [22], but are more likely due to the interpretation of that evidence base, and in fact may highlight the difference in mandates, methodology and reporting between and within regulatory and HTA agencies.

The aim of this analysis was to examine data at an aggregate level and to identify patterns in surrogate acceptability. It was outside of the scope and the objectives of this analysis to examine the merits of any individual drug or any individual recommendation. Furthermore, the analysis was restricted to the publicly available information. While this information is not comprehensive of the reimbursement evaluation and recommendation, it is nevertheless the only information available for external parties to analyze and interpret. As such, it has an inherent legitimacy in representing the reality known to the multitude of external stakeholders, rather than the few directly involved in the decision-making process.

A major challenge with this analysis was the subjectivity of data abstraction. Definitions and rules of hierarchy for surrogate acceptability were evolved for the analysis, as each additional recommendation added complexity to the task. This was further complicated by including recommendations from multiple agencies, with different reporting structures and expectations. This was evidenced by expanding the original binary categorization of accepted/non-accepted surrogate for the CDR-specific analysis to the eventual ten-level categorization of surrogate acceptability. Operationally, the categories expanded as subsequent recommendations presented fresh challenges; then, each previous recommendation had to be re-reviewed in the context of the evolving acceptability categorization. This greater complexity allowed better characterization of surrogate acceptability, although there were still challenging instances when both positive and negative comments were expressed in the same recommendation.

Surrogate outcomes have long posed problems to regulatory and reimbursement agencies, as well as to clinicians and clinical practice guideline working groups [7,9,23,24]. This challenge has only increased over time, as pressures to accelerate drug approval times has led to increasing use of surrogate outcomes – to the point where the surrogate itself may be posited as a meaningful outcome (such as progression free survival in cancer) [25,26]. Despite broad use and adoption of surrogate outcomes, unpredictable risks remain in assuming causal relationships between surrogates and clinical outcomes [27]. HTA agencies show appropriate caution when considering surrogates. Inevitably, however, reimbursement decisions must be made in the context of uncertainty, at an early point in a product’s life cycle, often with incomplete epidemiologic data and always with a lack of long-term data. The time lag in evidence development can be addressed with a number of different strategies, to better align the disparate informational needs of the HTA agency and the regulatory authority. Where the evidence is weak for a causal relationship between a surrogate and downstream clinical events, strategies to reduce uncertainty might include: early tripartite dialogue, coverage with evidence development, adaptive (progressive) licensing, epidemiological research and/or simple post-marketing randomized controlled trials [16].

Given the pivotal role of surrogate outcomes in drug development, and the observed variability in surrogate acceptability both within and between international agencies, more clarity is needed on the nature and timing of evidence needed to validate surrogate outcomes. This requires involvement from multiple stakeholders (researchers, industry, regulatory agencies, reimbursement agencies and other interested parties) to define and achieve adequate surrogate validation.

## Conclusions

While the majority of surrogate outcomes were accepted at the CDR, those that were non-accepted were significantly associated with clinical uncertainty and a DNL recommendation. There was inconsistency of surrogate acceptability across several international agencies. The findings of this study emphasize a need for clinical, regulatory, reimbursement and industry stakeholders to consider collaboratively establishing guidelines and principles on the use, validation, and acceptability of surrogates.

## Competing interests

All authors reported no conflicts of interest.

This research was partially funded by an unrestricted grant from Janssen Inc. This funding was not associated with any role in the design and conduct of the study, in the collection, management, analysis and interpretation of the data, or in the preparation, review or approval of the manuscript.

## Authors’ contributions

AR was responsible for the overall research design, data abstraction and descriptive analysis, and was the lead author on the manuscript. SJ and RH contributed statistical elements: methods, analysis and writing. RG contributed overall research design, statistical design and edited the manuscript. All authors read and approved the final manuscript.
